# NDG-CAM: Nuclei Detection in Histopathology Images with Semantic Segmentation Networks and Grad-CAM

**DOI:** 10.3390/bioengineering9090475

**Published:** 2022-09-15

**Authors:** Nicola Altini, Antonio Brunetti, Emilia Puro, Maria Giovanna Taccogna, Concetta Saponaro, Francesco Alfredo Zito, Simona De Summa, Vitoantonio Bevilacqua

**Affiliations:** 1 Department of Electrical and Information Engineering (DEI), Polytechnic University of Bari, Via Edoardo Orabona n.4, 70126 Bari, BA, Italy; 2 Apulian Bioengineering s.r.l., Via delle Violette n.14, 70026 Modugno, BA, Italy; 3 Laboratory of Preclinical and Translational Research, Centro di Riferimento Oncologico della Basilicata (IRCCS-CROB), Via Padre Pio n.1, 85028 Rionero in Vulture, PZ, Italy; 4 Pathology Department, IRCCS Istituto Tumori “Giovanni Paolo II”, Via O. Flacco n.65, 70124 Bari, BA, Italy; 5 Molecular Diagnostics and Pharmacogenetics Unit, IRCCS Istituto Tumori “Giovanni Paolo II”, Via O. Flacco n.65, 70124 Bari, BA, Italy

**Keywords:** nuclei segmentation, histopathology, deep learning, Grad-CAM, semantic segmentation, instance segmentation, nuclei detection

## Abstract

Nuclei identification is a fundamental task in many areas of biomedical image analysis related to computational pathology applications. Nowadays, deep learning is the primary approach by which to segment the nuclei, but accuracy is closely linked to the amount of histological ground truth data for training. In addition, it is known that most of the hematoxylin and eosin (H&E)-stained microscopy nuclei images contain complex and irregular visual characteristics. Moreover, conventional semantic segmentation architectures grounded on convolutional neural networks (CNNs) are unable to recognize distinct overlapping and clustered nuclei. To overcome these problems, we present an innovative method based on gradient-weighted class activation mapping (Grad-CAM) saliency maps for image segmentation. The proposed solution is comprised of two steps. The first is the semantic segmentation obtained by the use of a CNN; then, the detection step is based on the calculation of local maxima of the Grad-CAM analysis evaluated on the nucleus class, allowing us to determine the positions of the nuclei centroids. This approach, which we denote as NDG-CAM, has performance in line with state-of-the-art methods, especially in isolating the different nuclei instances, and can be generalized for different organs and tissues. Experimental results demonstrated a precision of 0.833, recall of 0.815 and a Dice coefficient of 0.824 on the publicly available validation set. When used in combined mode with instance segmentation architectures such as Mask R-CNN, the method manages to surpass state-of-the-art approaches, with precision of 0.838, recall of 0.934 and a Dice coefficient of 0.884. Furthermore, performance on the external, locally collected validation set, with a Dice coefficient of 0.914 for the combined model, shows the generalization capability of the implemented pipeline, which has the ability to detect nuclei not only related to tumor or normal epithelium but also to other cytotypes.

## 1. Introduction

In the healthcare scenario, artificial intelligence is exploited in medical imaging as a powerful tool with which to characterize objects of interest and lesions in anatomical regions under consideration. Traditionally, pathologists manually analyze numerous biopsies or tissue samples to diagnose complex pathologies, such as cancer. Even though it is tedious and time-consuming, this approach remains the gold standard [1,2].

Computational pathology attempts to overcome the main challenges arising from manual histological image evaluation, such as inter- and intraobserver variability or the inability to evaluate the smallest visual features and the time required to examine whole slide images (WSIs) [1,3,4].

The nuclei of cells provide a great deal of information for the analysis of histopathological tissue. For instance, immunohistochemistry-marked nuclei can be exploited for the estimation of cellular proliferation in cancer (e.g., Ki-67). Hence, nuclei segmentation is a fundamental first step toward the automated analysis of WSIs [5]. However, the difficulties associated with variable coloring arising from hematoxylin and eosin (H&E)-stained images, overlapped nuclei, the presence of artifacts, and differences in cell morphology and texture, represent obstacles for computer-based segmentation algorithms [2,3]. Moreover, WSIs have very high resolutions and contain an enormous number of nuclei, adding peculiarity to the task [6]. A critical aspect in several computational pathology pipelines is to achieve accurate segmentation of nuclei both for subsequent extraction and classification of nucleus features, but also for analyzing cellular distribution, useful for classifying tissue subtypes and identifying abnormalities [3].

Several studies focused on nuclei detection because of its importance in the pathologic diagnostic pipeline, in particular in the field of oncology. As an example, nuclei detection could be helpful to distinguish nuclei undergoing changes, indicating a progression of squamous epithelium cervical intraepithelial neoplasia [7]. Moreover, the estimation of tumor cellularity is very important, particularly in the era of precision medicine. Indeed, bioinformatic pipelines for copy number variation analysis require tumor cellularity as input and for a correct evaluation of variant allelic frequency [8].

Machine learning-based nuclear segmentation methods are typically the most efficient, as they can learn to identify variations in the shape and coloration of nuclei. In the semantic segmentation [9,10] approach, all image pixels are labeled as nuclear or background through a deep learning model. Nevertheless, these methods often fail to distinguish the different instances of objects of interest, i.e., nuclei, which then need to be addressed with ad hoc post-processing techniques, such as clustering [11].

The detection task can be approached by exploiting morphological features. CRImage [12] profits from thresholding as the first step for nuclei detection. Centroids of segmented nuclei are used as the point of detection. Then, a list of statistics for each segmented nucleus is utilized as a feature vector, and classification involves a support vector machine with radial basis kernel. Finally, spatial density smoothing is used to correct false detections.

LIPSyM [13] introduces the local isotropic phase symmetry measurement, designed to give high values to cell centers and nearby pixels; on the other hand, it cannot precisely detect spindle-like and other irregularly shaped nuclei such as fibroblasts and malignant epithelial nuclei.

In the last several years, convolutional neural networks (CNN) are emerging as the most effective way to tackle the nuclei detection task. In particular, the spatially constrained convolutional neural network (SC-CNN) [14] uses spatial regression for localizing the nuclei centers; the regression in SC-CNN is model-based, which explicitly constrains the output form of the network.

Xu et al. [6] used a stacked sparse autoencoder (SSAE) to learn a high-level representation of nuclear and non-nuclear objects by means of a softmax classifier.

Finally, the R2U-Net-based regression model named “UD-Net” [4] is proposed for end-to-end nuclei detection from pathological images. The recurrent convolutional operations help the model learn and represent features better than the feed-forward convolutional operations, and the robustness of the R2U-Net model has been demonstrated previously in several studies [15].

Methodologies prior to the advent of deep learning demonstrate worse performance on the nuclei detection task. Moreover, handcrafted feature extraction is a tedious and complex process, which can lead to different results depending on the experience of the feature engineers and domain experts. It is worth noting that CNN-based approaches require datasets with a distinct label for every nucleus, based on observations made in the last several years. Simple existing semantic segmentation methods, trained without the knowledge of different instances, cannot be reliably adopted for nuclei detection.

Many cell nuclei detection methods share a basic approach that includes generating an intermediate map through a CNN that indicates the presence of a nucleus, called the probability or proximity map (P-Map) [3,16], or have specialized architectures that are trained to individuate the centers of the nuclei, such as SC-CNN [14]. Indeed, the P-Map represents proximities as a monochromatic image: the intensities have high values near the centroid of the nucleus, and gradually lower going toward the boundaries.

By following the idea of determining a structure similar to a P-Map, we propose a novel method for nuclei detection, without the need for specialized architectures or handcrafted feature extraction; rather, only semantic segmentation networks and explainable artificial intelligence (XAI) techniques are used. The proposed method is quick to train, and is extensible because it can be plugged on top of existing semantic segmentation networks.

The presence of clustered or overlapped nuclei with semantic segmentation models can be spotted on visual inspection of the images. In order to overcome this issue, we exploited the potentialities of the gradient-weighted class activation mapping (Grad-CAM) for segmentation, which made it possible to highlight the activation of the nucleus class (compared to the background class), thus obtaining a saliency map with properties similar to the classic P-Map. The locations of the nuclei are subsequently determined by looking for local maxima in the activation map. Starting from the identified centroids, it is possible to associate all the pixels belonging to the considered nucleus, with a proximity criterion. This model alone, which we denote as nuclei detection with Grad-CAM (NDG-CAM), was capable of achieving performance in line with state-of-the-art methods. Because the Mask R-CNN [17] instance segmentation architecture is widely employed and constitutes a standard baseline for these tasks, we also realized a combined model for further enhancing the results, surpassing the state of the art.

To summarize, our contributions can be considered as follows: (i) we introduce a novel detection method for nuclei—NDG-CAM—which exploits Grad-CAM for semantic segmentation; (ii) we collected and annotated a local dataset of patients diagnosed with colorectal cancer to show the applicability of the proposed method in a local hospital; (iii) we examined and compared different state-of-the-art techniques to show the effectiveness of the proposed approach; (iv) we trained and evaluated an instance segmentation architecture as the baseline; and (v) we proposed a combined model which, exploiting both NDG-CAM and Mask R-CNN, can surpass the current literature performance concerning nuclei detection.

The remainder of the manuscript is organized as follows. Section 2 first describes the datasets adopted for the analysis. Then, semantic segmentation configurations and architectures are presented. The NDG-CAM is proposed, and its workflow is delineated. An instance segmentation is also considered as the baseline. Lastly, implementation details, the combined model, and the evaluation metrics employed for the analysis are presented. Results are portrayed in Section 3 and discussed in Section 4. A comparison with other state-of-the-art approaches is considered here. Lastly, final remarks, conclusions, and ideas for future works are drawn in Section 5.

## 2. Materials and Methods

### 2.1. Datasets

For the tasks of nuclei segmentation and detection, different datasets were considered in order to find the best-performing model. In particular, we considered the latest and largest publicly available datasets for nuclei detection and segmentation. Moreover, a local dataset has been collected, to prove the feasibility of the proposed system on new data from a local hospital.

- **MoNuSeg** [1,18,19]. The cell nucleus segmentation dataset used in this work is publicly accessible from the 2018 Data Science Bowl challenge [20]. The dataset contains a large number of segmented nuclei images and includes different cell types; there are 30 training H&E images containing 21,623 hand-annotated nuclear boundaries from the breast, kidney, prostate, liver, colon, bladder, and stomach. Moreover, there are also 14 H&E test images containing 7000 nuclear boundary annotations from the breast, kidney, prostate, colon, bladder, lung, and brain. All images, each of size 1000 × 1000, were captured at 40× magnification. The nuclear contour annotations are provided through XML files.
- **CRCHistoPhenotypes**: Labeled Cell Nuclei Data [14,21]. This publicly available dataset contains 100 H&E-stained histology images of colon cell nuclei obtained from WSI of 10 patients with a magnification factor of 20×. Tiles have a size of 500 × 500. Nuclear annotations are provided through the coordinates of the centroids in .mat format, resulting in a total of 29,756 annotated nuclei for detection purposes.
- **NuCLS** [22]. The dataset contains over 220,000 labeled nuclei from breast cancer images from TCGA, obtained from 125 patients with breast cancer (1 slide per patient) and captured with a magnification factor of 40×. These nuclei were annotated through the collaborative effort of pathologists, pathology residents, and medical students. Data from both single-rater and multi-rater studies are provided. For single-rater data, there are both pathologist-reviewed and uncorrected annotations. For multi-rater datasets, there are annotations generated with and without suggestions from weak segmentation and classification algorithms. We used only the single-rater dataset, which is already split into train and test sets. The annotations for the single-rater dataset include 59,485 nuclei and 19,680 boundaries, extracted from 1744 H&E image tiles of variable dimensions between 200 and 400 pixels.
- **Local dataset** from Pathology Department of IRCCS Istituto Tumori Giovanni Paolo II [23]. This consists of 19 H&E image tiles which overall contain more than 6378 nuclei from patients with colorectal cancer. Images have a size of 512 × 512 and were captured at 40× magnification. Annotations have been provided by a biologist with experience in analyzing histopathological data.

**Figure 1:**
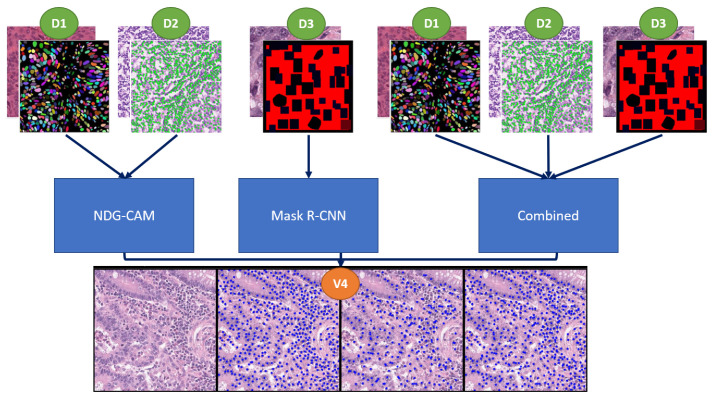
Pipeline adopted for training and validation. D1 and D2 datasets have been used to train and select the best semantic segmentation network. D3 dataset has been exploited to train the Mask R-CNN instance segmentation architecture. Finally, external validation has been conducted on the local validation dataset V4.

Hereafter, we will denote with T1 and V1 the training and test sets of MoNuSeg (D1), and with D2 the overall dataset of CRCHistoPhenotypes. The Mask R-CNN model has been trained on the NuCLS (D3) dataset, being the largest publicly available dataset with annotations formatted for instance segmentation. Because D1 already includes a validation set, we have used that one for the first validation stage. As an independent external validation set, we collected other image tiles from the Pathology Department of IRCCS Istituto Tumori Giovanni Paolo II [23], which will be denoted as V4, in order to assess the generalization capability of the best semantic segmentation network configuration individuated with the D1 and D2 datasets, and the Mask R-CNN model trained on the D3 dataset. Figure 1 summarizes the pipeline implemented for training and validating the models.

**Figure 2:**
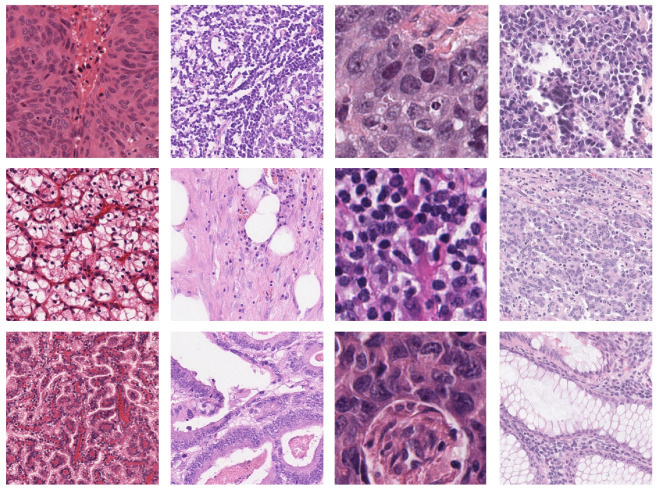
Sample images of datasets for nuclei detection. (First column) D1—MoNuSeg [18]; (second column) D2—CRCHistoPhenotypes [21]; (third column) D3—NuCLS [22]; (fourth column) V4—local dataset [23].

A summary of the details for the employed datasets is reported in Table 1, whereas sample images are depicted in Figure 2.

### 2.2. NDG-CAM

In this section, we introduce the methodology adopted for NDG-CAM. Several steps have been carried out. As the first step, a semantic segmentation architecture trained for nuclear segmentation is required. Different experimental configurations of the datasets and network architectures have been compared in order to find the most suitable model, with details reported in Section 2.2.1 and Section 2.2.2. Then, the Grad-CAM technique for semantic segmentation, which is still underexplored if compared to Grad-CAM for classification, has been employed to obtain saliency maps of the nuclei, with higher values of intensity corresponding to positions nearest to the centroids. Subsequently, a search for local maxima, combined with post-processing and clustering, allowed for the detection and eventually instance segmentation of the nuclei. This process is presented in Section 2.2.3. Compared to specialized architectures, such as those used for instance segmentation, semantic segmentation networks are simpler and faster to train. In addition, our system can be trained if labels do not distinguish between different nuclear instances, which would not be possible for instance segmentation models.

#### 2.2.1. Semantic Segmentation Workflow

Starting from the datasets described in the previous sections, the following experiments were carried out, all with images at a size of 512 × 512:

a Train on D2 and validation on V1 at 20× resolution.
b Train on T1 and validation on V1 at 20× resolution.
c Train on T1 and validation on V1 at 40× resolution.

In the first two experiments, images were padded from 500 × 500 to 512 × 512 exploiting the mirror padding. Instead, in the last experiment, the images were padded from 1000 × 1000 to 1024 × 1024 with mirror padding and subsequently divided into 4 tiles of 512 × 512. For each experiment, different deep network architectures were trained and compared: U-Net [24], SegNet [25], and DeepLab v3+ [26] in three different backbone configurations, namely ResNet18, ResNet50 [27], and MobileNet-v2 [28]. The aforementioned experiments were carried out in MATLAB R2021a.

#### 2.2.2. Network Architectures

The segmentation phase is a milestone for the detection phase; this step aims to discriminate between cell nuclei and the background. semantic segmentation architectures play a role of pivotal importance in deep learning-based medical image analysis [9,29,30,31]. It is a process that associates a label or a category to each pixel of an input image, thus allowing the pixelwise spatial localization of each object category appearing in the scene.

In the specific case under analysis, the goal was to segment the cell nuclei in a robust way, so as to provide satisfactory results even when the algorithm would have been applied to different images of the same type. For this reason, it was decided to carry out the same experiments with several convolutional architectures.

The considered architectures include:

- U-Net [24]. It is a fully convolutional network to perform the semantic segmentation task. The U-Net architecture consists of a series of encoding layers and contractions that are used to extract the context of the image, followed by a sequence of symmetrical decoding layers and expansions to recover the spatial information. In our MATLAB setting, the network is characterized by 58 convolutional layers; the first layer deals with a z-score normalization of the inputs, whereas the last one presents the Dice function as a loss function.
- SegNet [25]. This is another encoder–decoder architecture. In this case, the decoding blocks exploit max pooling indices received from the corresponding contraction block to perform the oversampling, instead of using trainable upsampling layers as transposed convolutions. In our MATLAB setting, this CNN consists of 31 layers with a cross-entropy loss function.
- DeepLab v3+ [26]. This architecture features atrous spatial pyramid pooling (ASPP) and the encoder–decoder paradigm. The first aspect concerns a particular way of combining layers of atrous and depthwise convolution, with which the model captures and concatenates features at different scales. For this network, the backbone is customizable. Three different basic CNN encoders were used: ResNet18, ResNet50, and MobileNet-v2. The DeepLab v3+ has 100 layers, of which the last is a softmax layer that is used to obtain the probabilities that each pixel belongs to the nucleus or background class; in this case, the chosen loss function is the Dice loss.

**Figure 3:**
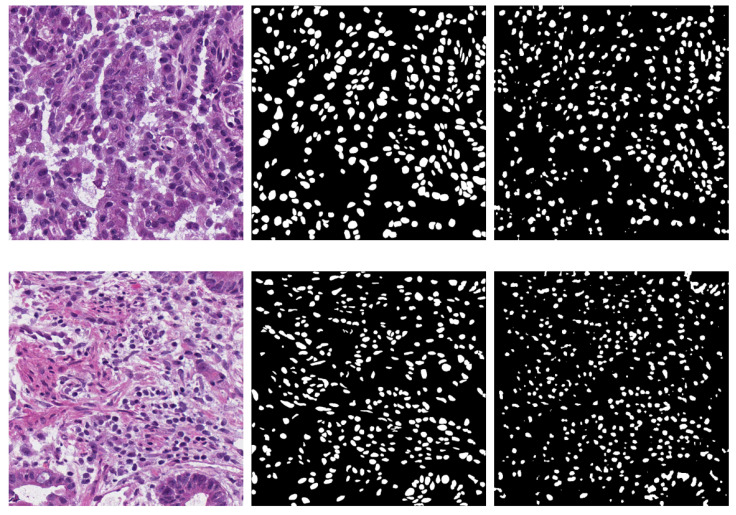
Semantic segmentation output for nuclei images. (**Left**) Original image. (**Middle**) Ground truth. (**Right**) Prediction of experiment (b) with DeepLab v3+ and backbone ResNet18.

An example of semantic segmentation prediction from DeepLab v3+ with backbone ResNet18 is shown in Figure 3.

#### 2.2.3. Nuclei Detection with Grad-CAM

After the best performing network has been identified, the output returned by the semantic segmentation was a mask in which the pixels of the input image were classified into pixels belonging to the foreground, i.e., nucleus, or background class. As mentioned previously, this did not allow us to distinguish multiple instances of the same object and therefore to distinguish multiple nuclei adjacent to each other.

In this scenario, the detection phase begins. In fact, after the semantic segmentation, post-processing was carried out in order to solve this problem. The first step was to calculate the Grad-CAM of the input image according to the chosen network. A CNN is often seen as a black box, or rather, as a model with parameters *W* that, given an image of input *X*, through a function f(X,W), is able to map to the related output *y*. XAI techniques have been designed in order to unveil the underlying mechanisms involved in the processing stages of deep neural networks, and are recently gaining a lot of attention in medical imaging and clinical decision support systems [32,33,34,35].

During the training phase, even if we are capable of achieving high performance according to the considered metrics, we do not know which image features are more determinant for the network to make its choices. One of the ways to visually solve this problem is Grad-CAM [35].

Grad-CAM is typically used in image-classification scenarios [36], but it can also be extended to semantic segmentation problems [37]. In general, the heatmap $L^{c}$ for class *c* is generated by using $a_{c}^{k}$ (as defined in Equation (1)) to sum the feature maps $A^{k}$, as in Equation (2).

(1) $$a_{c}^{k}=\frac{1}{N}\sum_{u,v}\frac{\partial y^{c}}{\partial A_{uv}^{k}}$$

(2) $$L^{c}=\mathrm{ReLU}\left(\sum_{k}a_{c}^{k}A^{k}\right)$$

*N* is the number of pixels and (u,v) are the indices. ReLU is applied pixelwise to clip negative values at zero, to only highlight areas that positively contribute to the decision for class *c*. The difference with the classification task is that for semantic segmentation $y^{c}$, the scalar class score, is obtained by reducing the pixelwise class scores for the class of interest to a scalar [37], as in Equation (3).

(3) $$y^{c}=\sum_{(u,v)\in P}Y_{\left(u,v\right)}^{c}$$

*P* is a set of pixel indices of interest in the output layer: in our case, the softmax layer before the pixel classification layer. Higher values of $L^{c}$ map indicate which areas of the image are important for the decision to classify pixels.

**Figure 4:**
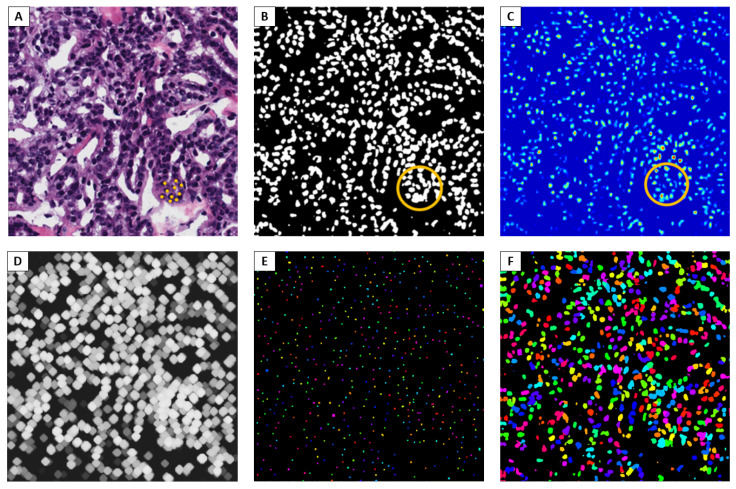
NDG-CAM Detection workflow. (**A**) Zone with multiple neighboring instances of nuclei. (**B**) Failure to recognize adjacent nuclei. (**C**) Grad-CAM for semantic segmentation. (**D**) Dilated image. (**E**) Connected components. (**F**) Detection prediction.

In the proposed approach, the activation of the network for the nucleus class was analyzed, obtaining a probability map with values that we denote as CAM-Map. Therefore, activations greater in correspondence with the centroids of the nuclei (even when adjacent to each other) are visible from Figure 4C.

From CAM-Map, we applied a morphological grayscale dilation operator with a spherical shape factor of radius 7. The result is depicted in Figure 4D. This step allowed the enlargement of the activation areas so that no false nuclei were identified in the nearby regions where activations were not high enough compared to the maximum point.

Then, as portrayed in Figure 4E, we proceeded with the calculation of the local maximum of the regions and the localization of all the connected components, with the related geometric centroids, which correspond to the identified nuclei.

Once the centroids were found, K-means clustering, with K equal to the number of connected components, has been exploited to associate the adjacent pixels to each nucleus, so as to have the overall predicted mask of the original starting image. The final mask is reported in Figure 4F.

### 2.3. Instance Segmentation

Object detection involves the detection, with a bounding box, of all the different objects of interest present in a scene. Instance segmentation further extends this task, by also considering the problem of delineating a precise mask around each object. Architectures for object detection are usually divided into one-stage and two-stage models, with the first being faster and the former being more accurate. Inside the realm of methods for two-stage object detectors, a pivotal role has been played by architectures from the R-CNN family [38].

Mask R-CNN evolves the R-CNN family by adding a semantic segmentation branch, making the model capable of performing instance segmentation [17]. The overall Mask R-CNN architecture is composed of two parts: the backbone architecture, which performs feature extraction, and the head architecture, which performs classification, bounding box regression, and mask prediction.

We employed the Detectron2 [39], a platform powered by the Pytorch framework, that provides state-of-the-art detection and segmentation algorithms. It includes high-quality implementations of the most popular object detection algorithms, comprising different variants of the pioneering Mask R-CNN model. Detectron2 has an extensible design so that it can be easily employed to implement cutting-edge research projects.

The NuCLS dataset [22] was chosen to train the network, the instance segmentation model mask_rcnn_R_50_DC5_1x. Annotations were converted into the COCO annotation format for adoption in the Detectron2 framework.

### 2.4. Implementation Details

All the semantic segmentation networks have been trained on a laptop with a GeForce GTX960M. For carrying out the training, the chosen optimizer was SGDM, with a starting learning rate of 0.05. The learning rate schedule was piecewise with a drop factor of 0.94 and a drop period of 2. $L_{2}$ regularization parameter was set to 0.0005. With a batch size of 2, 15 epochs lasted roughly 105 min for the best performing architecture, DeepLab v3+ with ResNet18 as the backbone.

The Mask R-CNN model, being heavier, has been trained on a Google Colab Pro environment. With a Tesla P100, 20,000 iterations were carried out in roughly 110 min. The chosen optimizer was SGDM, as set by default in the Detectron2 environment, with a starting learning rate of 0.00025.

### 2.5. Combined Model

In order to obtain the advantages of both approaches, a combined model has been developed.

It exploits a criterion for obtaining merged outputs from NDG-CAM detection and Mask R-CNN. In detail, a distance criterion was used to check if a nucleus was found by only one of the approaches. In that case, the nucleus was simply retained. Instead, if more nuclei centroids are found in proximity, only the ones found by Mask R-CNN are retained. The combined methodology has the idea to increase the recall, which is very important because nuclei detection is the first stage for further analyses.

### 2.6. Evaluation Metrics

Each semantic segmentation architecture described in Section 2.2.1 was tested in all three experimental configurations mentioned. In order to assess the goodness of pixelwise classification performed by semantic segmentation networks, the pixelwise precision, recall, and Dice coefficient were considered as performance indices. Given pixelwise true positives (TP), false positives (FP) and false negatives (FN), then precision, recall, and Dice coefficient can be defined as in Equation (4), Equation (5), and Equation (6), respectively:

(4) $$Precision=\frac{TP}{TP+FP}$$

(5) $$Recall=\frac{TP}{TP+FN}$$

(6) $$Dice=\frac{2\cdot TP}{2\cdot TP+FP+FN}.$$

For all these metrics, a higher value denotes a better segmentation result; that is, predicted masks are more similar to ground truth ones.

**Figure 5:**
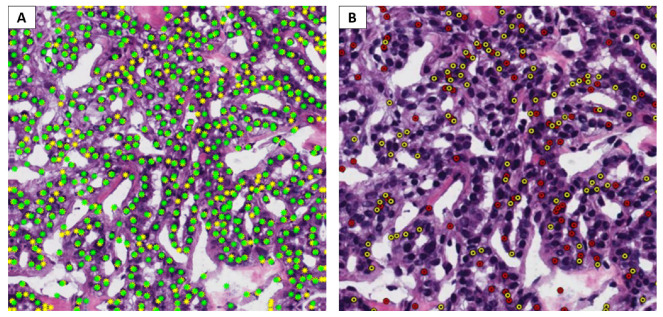
Example of calculation of evaluation metrics for object detection. (**A**) Prediction vs ground truth. Yellow, ground truth; green, prediction; (**B**) Differences between prediction and ground truth. Yellow, detection FN; red, detection FP.

Instead, for assessing the detection procedure, we considered two kinds of metrics. The first is based on the simple calculation of the number of detected nuclei with respect to the ground truth. The error ($e_{a}$), defined in Equation (7), is given by the difference in absolute value between the number of nuclei found and the real number, divided by the latter. An example of the prediction vs. ground truth result, which is the basis for enumerating nuclei, is depicted in Figure 5A. Because we were also interested in understanding if our algorithm was more prone toward overdetection or underdetection, a signed error ($e_{s}$), defined in Equation (8), was also evaluated:

(7) $$e_{a}=\frac{\left|d-g\right|}{g}$$

(8) $$e_{s}=\frac{d-g}{g}.$$

In these two equations, *d* denotes the number of detected nuclei, whereas *g* is the number of ground truth nuclei.

The second category of metrics includes Dice coefficient, precision, and recall for object detection, which can provide more information about the quality of the detection results. In this case, we are not simply rewarding our prediction of as many nuclei as are present in the ground truth, but we also want to ensure that detected nuclei are in the right place. In order to achieve this result, we need to discover object detection FP and FN, as can be seen in Figure 5B. In order to determine these quantities, as the first step, we computed the distance matrix between the centroids of the detected nuclei and the real ones. In order to decide whether a detection actually corresponds to a nucleus centroid, a distance threshold ξ was considered, equal to the mean radius of the nuclei of each image [16]. If the distance between a prediction and a ground truth annotation is less than or equal to ξ, the prediction is counted as a TP. If more than one detection verifies this condition, the one closest to the ground truth position is counted as TP and the others as FP. The detections further than ξ from any ground truth location are counted as FP, and all ground truth annotations without close detections are marked as FN. Lastly, the following control condition was added. If the distance between an FP and an FN is less than an ϵ threshold, set to 6 (a value close to the nuclear radius), the count of FP and FN will each be decreased by one, whereas TP will be increased by one. The pseudocode for determining TP, FP, and FN is reported in Algorithm 1.

In order to assess the statistical significance of the obtained results calculated per case, we determined the *p*-value with the two-tailed Wilcoxon signed-rank test. The threshold for significance has been set to 0.05.
**Algorithm 1:** Object Detection TP, FP, FN calculation.
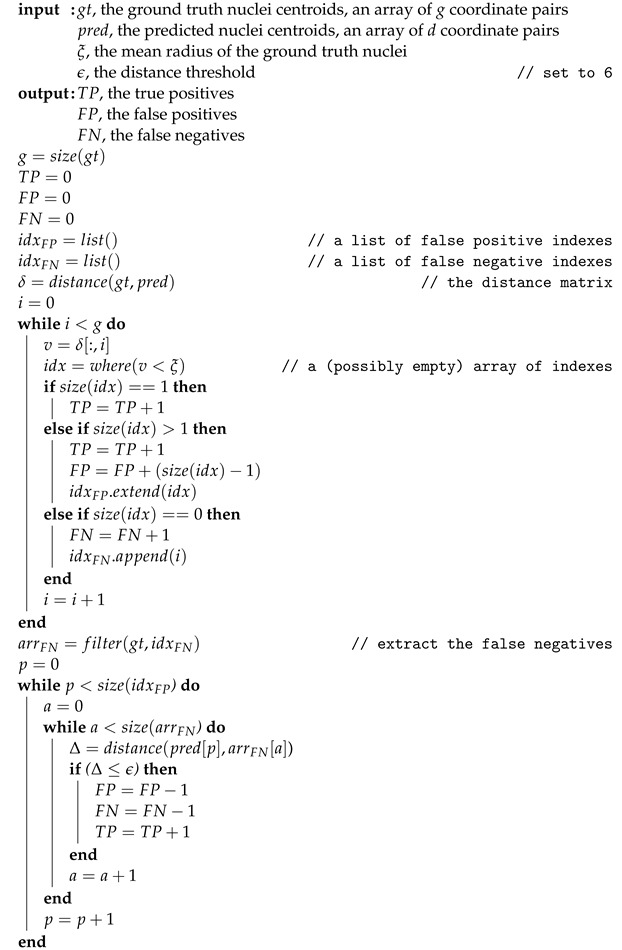


## 3. Results

The automatic segmentation of cell nuclei attracted significant interest from the scientific community, as their identification is an important starting point for many medical analyses based on histopathological images. In this work, for the semantic segmentation phase, different architectures were elaborated and tested on different datasets, for a total of 15 experiments. For each of them, performance indices were calculated to identify the best model with which to proceed for the subsequent phases. From this comparison, it emerged that the best performance can be obtained by referring to the experimental configuration (b) defined in Section 2.2.1.

Table 2 reports the results obtained for each network architecture in the semantic segmentation task. For DeepLab v3+, the backbone architecture is included within square brackets.

It therefore emerges that the best solution coincides with experiment (b) conducted with DeepLab v3+ using the ResNet18 network as the backbone. It allowed us to obtain a pixelwise Dice coefficient of 74.23 ± 4.85%, a precision of 76.42 ± 8.69%, and a recall of 74.25 ± 11.23%.

DeepLab v3+ was hence chosen as the base model to be exploited in the detection phase. By exploiting the Grad-CAM for semantic segmentation, it was possible to retrieve nuclei centroids via local maxima of the obtained saliency maps.

On the V1 dataset, the experimental results demonstrated an $e_{a}$ of the identified nuclei equal to 2.11%, 2.43%, and 11.50% for the NDG-CAM, Mask R-CNN, and combined method, respectively. When calculated per case, the values for $e_{s}$ were 1.84 ± 13.05%, 3.46 ± 6.15%, and 14.45 ± 11.22%, indicating that the models generally tend to overdetect on this dataset.

In the V4 dataset, the $e_{a}$ had a value of 15.26%, 59.22%, and 14.10% for the NDG-CAM, Mask R-CNN, and combined method, respectively. When calculated per case, the values for $e_{s}$ were −16.86 ± 13.79%, −60.13 ± 13.88%, and −14.88 ± 12.86%, showing that the models have a tendency to underdetect on this dataset. In particular, it was noticed that very small nuclei, such as those of lymphocytes, and elongated ones, such as those of fibrocytes, were underdetected.

For the detection task, the results are reported in Table 3. In the V1 dataset, NDG-CAM, Mask R-CNN, and the combined method were capable of achieving a Dice coefficient of 0.824, 0.878, and 0.884, respectively. Thus, the combined method obtained slightly better results than the other methods. As for the recall, the combined method decisively surpasses the other approaches, with a value of 0.934.

In the V4 dataset, the combined method proves to be the best, achieving a recall of 0.850 and a Dice coefficient of 0.914. Mask R-CNN performs poorly in this case, with a recall of 0.403 and a Dice coefficient of 0.573.

**Figure 6:**
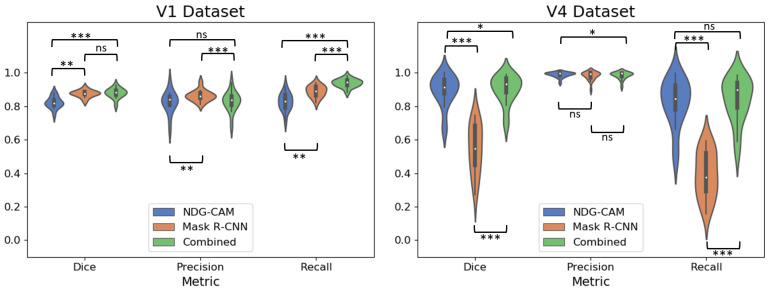
Violin plots for the detection metrics calculated per case. (**Left**) V1 dataset. (**Right**) V4 dataset. In the figure, ns stands for nonsignificant; * denotes *p*-value < 0.05; ** indicates *p*-value < 0.01; and *** means *p*-value < 0.001.

The violin plots calculated per tile are reported in Figure 6 for the V1 and V4 datasets, comparing the NDG-CAM detection method, Mask R-CNN, and the combined approach. It is worth noting that the Mask R-CNN model works very well on the V1 dataset but performs poorly on the V4 one. On the other hand, the NDG-CAM and the combined methods maintain high levels of performance in all scenarios.

In the V1 dataset, the combined model does not show a Dice coefficient that is higher in a statistically significant way than the Mask R-CNN approach, with a *p*-value of 0.07. On the other hand, the recall was much higher for the combined method, resulting in a *p*-value < 0.001 for both NDG-CAM and Mask R-CNN. In the V4 dataset, both the NDG-CAM and the combined method showed much stronger results than Mask R-CNN, with a *p*-value less than 0.001 in both cases for Dice coefficient and recall. Moreover, the combined approach shows a statistically significant advantage over NDG-CAM (*p*-value = 0.048) for the Dice coefficient.

## 4. Discussion

In order to show the effectiveness of the proposed method, we compared it with existing state-of-the-art approaches. It has to be noted that our method allows exploiting semantic segmentation architectures to realize nuclei detection, whereas other approaches usually involve networks specialized for this task. Several approaches proposed in the literature try to localize centers of the nuclei or proximity maps to those centers [3,14,16]. These approaches require instance-level annotations, although the results are promising. On the other hand, the proposed method exploits an XAI technique, Grad-CAM for semantic segmentation, to reconstruct post hoc saliency maps that are related to the centers of the nuclei, showing that semantic segmentation networks can perform detection tasks without specialized modifications.

The most widespread metrics employed for assessing algorithms for object detection involve precision, recall, and Dice coefficient. Namely, they are the metrics that are also related to the position of the detected nuclei, and not only on the counts.

A quantitative comparison between considered approaches and existing ones from the literature is presented in Table 3.

From this comparative analysis, it emerges that the proposed method is perfectly aligned with the state of the art, without the need to implement specific kinds of specialized loss functions [24] or architectures for detection [17,40].

Indeed, the NDG-CAM method alone was capable of achieving a Dice coefficient for object detection of 0.824, whereas the UD-Net [4] method, the top-performing method among the selected from the literature, had a Dice coefficient of 0.828. When the proposed NDG-CAM detection method is used in combined usage with Mask R-CNN, the recall increases to 0.934, and the Dice coefficient to 0.884, surpassing the current state-of-the-art methods for nuclei detection. On the collected external validation set, metrics are even higher, with a Dice coefficient of 0.914, showing the generalization capabilities of the proposed workflow.

**Figure 7:**
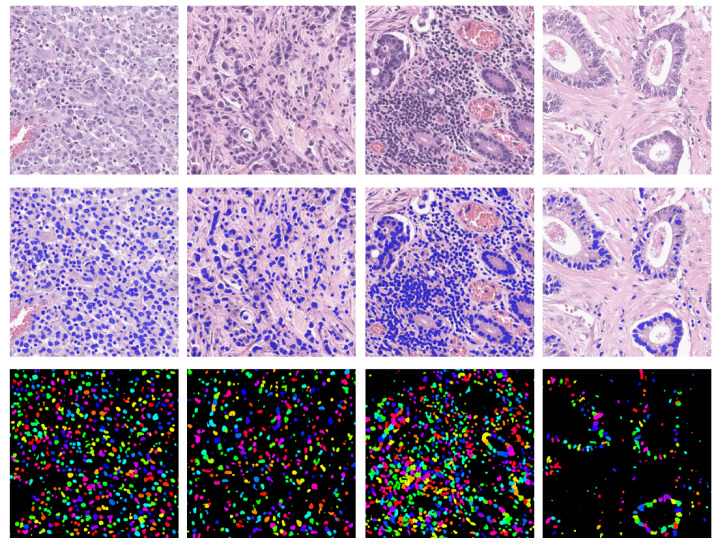
Examples of the NDG-CAM method on the data from the Pathology Department of IRCCS Istituto Tumori Giovanni Paolo II. Results are shown for the best architecture (DeepLab v3+ with ResNet18 backbone). (**First row**) Original images. (**Second row**) Semantic segmentation. (**Third row**) Instance segmentation after detection of centroids of the nuclei, with each color denoting a different nuclear instance.

**Figure 8:**
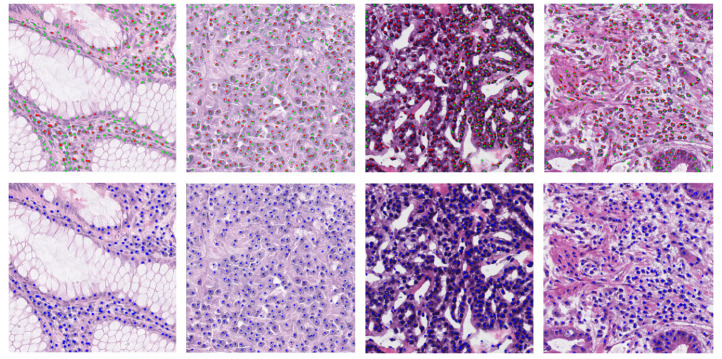
Examples of centroid detection on the validation sets V1 and V4. (**Top row**) Green, NDG-CAM method detections; red, Mask R-CNN detections. (**Bottom row**) Blue, combined method detections. First and second columns show data from V4, whereas the third and fourth columns depict data from V1.

Qualitative results for the the object detection pipeline involving semantic segmentation and Grad-CAM on the images of the independent external validation set V4 are depicted in Figure 7. Instead, Figure 8 shows the final detection results on the validation datasets V1 and V4 with the NDG-CAM method, the Mask R-CNN architecture, and the combined adoption of both methods.

It can be seen from the images of Figure 7, taken from the V4 dataset, that precision is very high. Indeed, virtually all detected nuclei are real. Some small or elongated nuclei, such as lymphocytic or fibrocytic nuclei, are underdetected. This may be due to a lack of proper training datasets with a large variety of nuclear shapes.

The two methods show similar performance on the V1 dataset, as can be observed from Figure 8. Mask R-CNN achieves slightly better performance on this dataset, and considering that it has been trained on a larger training set, the combined method proved to be superior. From the same figure, it is possible to observe that, in the V4 dataset, Mask R-CNN does not properly generalize, resulting in the missing of many nuclei (low recall).

## 5. Conclusions and Future Works

In this work, a novel method was presented with the aim of nuclei identification from histological H&E images. In our multi-stage pipeline, the first phase involved semantic segmentation. After various experiments, DeepLab v3+ (ResNet18 backbone) emerged as the best-performing architecture. Subsequently, because this analysis did not allow the distinction of multiple instances of the same object, we proposed a novel detection algorithm, NDG-CAM, which exploited Grad-CAM to solve the problem of separating the instances. Even without the need to use specialized loss functions or architectures, it allowed us to achieve satisfactory results in the detection task, comparable to or even better than more sophisticated training setups [3,6,12,16]. When the method is combined with the Mask R-CNN instance segmentation architecture, results exceed the state-of-the-art methods for nuclei detection.

Even though the local validation set includes only colorectal cancer H&E slides, it has to be considered that in each slide there are several tissue types present (e.g., stroma, immune infiltration) and the proposed method has the ability to detect nuclei not only related to the tumor or normal epithelium of colon but also to other cytotypes.

Indeed, we noticed underdetection of lymphocytic or fibrocytic nuclei, and this could be explained by a lack of datasets enriched in these nuclei subtypes. For such a reason, a direction for future works includes the collection of a dataset with multiple and balanced nuclei annotations.

On the clinical side, the proposed workflow could be a valid tool to support pathologists in the detection and reporting of histological samples, thus allowing a considerable saving of time and resources, besides providing an objective tool that is more reliable than manual assessment. Future works will concern the classification of the detected nuclei, in order to estimate how many are malignant or subjected to specific lesions, so that important clinical parameters, such as neoplastic cellularity, can be determined quantitatively.

## Figures and Tables

**Table 1:** Summary of datasets for nuclei.

Dataset	Publication Year	Organs	Resolution	Number of H&E images	Number of Nuclei	Size (pixels)	Annotations Format
MoNuSeg—Train (T1) [1]	2017	breast, kidney, prostate, liver, colon, bladder, stomach	40×	30	21,623	1000 × 1000	Nuclei Contours
MoNuSeg—Test (V1) [1]		breast, kidney, prostate, colon, bladder, lung, brain		14	7000		
CRCHistoPhenotypes (D2) [14]	2016	colon	20×	100	29,756	500 × 500	Nuclei Centroids
NuCLS (D3) [22]	2019	breast	40×	1744	59,485	200–400 per side	Nuclei Contours or Bounding Boxes
Local (V4)	2022	colon	40×	19	6378	512 × 512	Nuclei Centroids

**Table 2:** Performance comparison between considered network architectures for semantic segmentation.

Network	Metric	Experiment (a)	Experiment (b)	Experiment (c)
**U-Net**	**DICE** **PRECISION** **RECALL**	66.74 ± 3.44 57.13 ± 8.15 83.56 ± 10.61	65.71 ± 8.57 52.69 ± 11.96 91.65 ± 6.57	60.74 ± 11.65 45.43 ± 11.77 96.46 ± 2.44
**SegNet**	**DICE** **PRECISION** **RECALL**	56.44 ± 9.31 67.09 ± 8.01 52.60 ± 16.20	65.05 ± 6.32 58.93 ± 14.23 81.35 ± 17.69	62.02 ± 12.28 51.67 ± 14.96 85.05 ± 13.24
**DeepLab v3+ [ResNet18]**	**DICE** **PRECISION** **RECALL**	52.21 ± 11.99 76.78 ± 6.60 41.76 ± 13.55	**74.23 ± 4.85** 76.42 ± 8.69 74.25 ± 11.23	72.17 ± 8.03 62.76 ± 11.78 87.17 ± 5.64
**DeepLab v3+ [ResNet50]**	**DICE** **PRECISION** **RECALL**	57.87 ± 6.88 59.70 ± 6.35 57.10 ± 10.43	61.68 ± 8.75 63.69 ± 7.51 60.71 ± 11.94	65.98 ± 7.84 54.14 ± 13.81 90.95 ± 10.02
**DeepLab v3+ [mobilenetv2]**	**DICE** **PRECISION** **RECALL**	56.64 ± 6.60 66.49 ± 5.56 50.66 ± 10.50	73.01 ± 7.56 73.50 ± 11.76 75.07 ± 10.38	66.31 ± 13.80 57.52 ± 16.31 85.35 ± 9.43

**Table 3:** Comparison of detection methods, extending the one proposed by Alom et al. [4] and Sirinukunwattana et al. [14].

Method	Precision	Recall	Dice
CRImage [12]	0.657	0.461	0.542
CNN [12]	0.783	0.804	0.793
SSAE [6]	0.617	0.644	0.630
LIPSyM [13]	0.725	0.517	0.604
SC-CNN (M = 1) [14]	0.758	0.827	0.791
SC-CNN (M = 2) [14]	0.781	0.823	0.802
UD-Net [4]	0.822	0.842	0.828
NDG-CAM (V1)	0.833	0.815	0.824
NDG-CAM (V4)	**0.992**	0.841	0.910
Mask R-CNN (V1)	**0.867**	0.888	0.878
Mask R-CNN (V4)	0.989	0.403	0.573
Combined (V1)	0.838	**0.934**	**0.884**
Combined (V4)	0.986	**0.850**	**0.914**

## Data Availability

The MoNuSeg [18], CRCHistoPhenotypes [21], and NuCLS [22] datasets are publicly available. The local dataset from IRCCS Istituto Tumori Giovanni Paolo II presented in this study is available upon request from the corresponding author.
